# Postplacental Placement of Intrauterine Devices: Acceptability, Reasons for Refusal and Proposals to Increase its Use

**DOI:** 10.1055/s-0041-1725053

**Published:** 2021-04-15

**Authors:** Maria Beatriz de Paula Leite Kraft, Mariana Miadaira, Marcos Marangoni, Cássia Raquel Teatin Juliato, Fernanda Garanhani Surita

**Affiliations:** 1 Department of Obstetrics and Gynecology, School of Medical Science, Universidade Estadual de Campinas, Campinas, SP, Brazil

**Keywords:** postpartum, contraception, intrauterine device, health education, pós-parto, contracepção, dispositivo intrauterino, educação em saúde

## Abstract

**Objective** To evaluate the acceptability of postplacental placement of intrauterine devices (PPIUD), reasons for refusal and suggested policies to increase its use.

**Methods** Cross-sectional study conducted at the Women Hospital of the Universidade de Campinas, Campinas, SP, Brazil. Postplacental placement of intrauterine devices was offered to women admitted in labor who did not present infections, uterine malformation, twin pregnancy, preterm birth, and were at least 18 years old. In case of refusal, the parturient was asked to give their reasons and the answers were classified as misinformation about contraception or other reasons. The following were considered misinformation: fear of pain, bleeding, contraception failure and future infertility. Bivariate analysis was performed.

**Results** Amongst 241 invited women, the refusal rate was of 41.9%. Misinformation corresponded to 50.5% of all refusals, and the reasons were: fear of pain (39.9%); fear of contraception failure (4.9%); fear of bleeding (3.9%); fear of future infertility (1.9%); other reasons for refusal were 49.5%. Parturients aged between 18 and 27 years old refused the PPIUD more frequently due to misinformation (67.4%), and older parturients (between 28 and 43 years old) refused frequently due to other reasons (63.6%) ( *p* = 0.002). The mean age of those who declined the PPIUD due to misinformation was 27.3 ± 6.4 years old, while those who declined for other reasons had a mean age of 29.9 ± 5.9 years old ( *p* = 0.017).

**Conclusion** The refusal of the PPIUD was high, especially amongst young women and due to misinformation. It is necessary to develop educative measures during antenatal care to counsel women about contraception, reproductive health and consequences of unintended pregnancy.

## Resumo

**Objetivo** Avaliar a taxa de aceitação do dispositivo intrauterino pós-placentário (DIUPP); os motivos de recusa e propor medidas que aumentem sua aceitação.

**Métodos** Estudo de corte transversal realizado no Hospital da Mulher da Universidade Estadual de Campinas, Campinas, SP, Brasil. O DIUPP foi oferecido a mulheres admitidas em trabalho de parto que não apresentavam: infecções, malformação uterina, gravidez gemelar, parto prematuro e com idade mínima de 18 anos. Em caso de recusa, perguntou-se o motivo, e as respostas foram agrupadas em informações equivocadas sobre contracepção ou outros motivos. Considerou-se informação equivocada: medo de dor, sangramentos, falha da contracepção e prejuízo da fertilidade. Análises bivariadas foram realizadas.

**Resultados** Entre 241 mulheres, a taxa de recusa foi de 41,9%. A desinformação correspondeu a 50,5% de todos os motivos de recusa, que foram: medo da dor (39,9%); medo da falha da contracepção (4,9%); medo de sangramento (3,9%), medo de o dispositivo intrauterino (DIU) prejudicar a fertilidade (1,9%). Outros motivos de recusa atingem 49,5%. Parturientes com idade entre 18 e 27 anos recusaram o PPIUD com mais frequência devido a desinformação (67,4%), e as mais velhas (com idade entre 28 e 43 anos) recusaram com frequência devido a outros motivos (63,6%) ( *p* = 0,002). Houve diferença entre a idade média de quem recusou o PPIUD por desinformação (27,3 ± 6,4 anos) em comparação com outras razões (29,9 ± 5,9 anos), ( *p* = 0,017). Além disso, ambos os grupos apresentaram altas taxas de recusa por desinformação, de 67,4 e 36,4%, respectivamente.

**Conclusão** A recusa do DIUPP foi alta, principalmente entre as mulheres jovens e por desinformação. Diante disso, é necessário o desenvolvimento de medidas educativas durante o pré-natal e aconselhar as mulheres sobre contracepção, saúde reprodutiva e gravidez indesejada.

## Introduction

Contraception is important to women as it allows them to decide whether it is the right time to conceive. In 2012, the occurrence rate of unintended pregnancies worldwide was of 53 for every 1,000 women aged between 15 and 44 years old, and its prevalence was of 40%. 1 In the United States of America (USA), 50% of all pregnancies were unplanned 2 and, in Brazil, the rate is 55.4%. 3 Also, in some Brazilian regions, this figure rises to 65% (such as in the South). 4 Amongst Brazilian women in the postpartum period, 25.5% reported feeling embarrassed to have conceived. 3 These statistics reflect the importance of assuring contraception to all women. 5 The postpartum period is a great opportunity to address contraceptive needs.

Nowadays, the most efficient contraceptive methods are long-acting reversible contraceptives (LARCs), including intrauterine devices (IUD) (copper and levonorgestrel [LNG] intrauterine systems) and subdermal progestin implants. They demand no changes in habit, are well tolerated, and are more effective than other methods with < 2 pregnancies in 1,000 users. 6 Also, LARC methods have the lowest discontinuation rates. 6 Still, short-term reversible methods are highly prescribed, 7 even though studies show low continuation rates. 8

Intrauterine devices must be offered to all women in reproductive age, especially after delivery, both vaginal and at the time of a cesarean delivery. In the USA, a study has shown that 35% of all pregnancies were accounted within 18 months after a previous pregnancy. Those pregnancies are more common among adolescent girls and are more likely to have been unplanned. 9 The postpartum period is an opportunity to counsel women about contraception because, at that time, women often do not plan to conceive again in the near future. However, it is known that ~ 40% of women do not attend medical appointments in the postpartum period and that of all women after childbirth that are nursing, 20% will ovulate again as early as in the 3rd month after parturition and, therefore, will be at risk of conceiving again. 10

The period immediately after childbirth is a great opportunity to provide contraceptive methods, including LARCs. 11 12 13 Therefore, it is important to evaluate the acceptability and refusal rates for these kinds of contraceptives, as well as the reasons for refusals, to create policies that stimulate women to adhere to contraception immediately after childbirth. The present study aims to evaluate the acceptance of PPIUD. Also, it is necessary to examine the refusal rate, the motives for refusal and the age of the patient at the time. These data are important to help in the creation of policies that could increase the acceptance of contraception immediately after childbirth.

## Methods

The present study was approved by the Ethical Committee of the Universidade de Campinas' (under number 80620717.6.0000.5404) and is part of a large group of studies that analyze the insertion of IUDs immediately after childbirth.

The data presented in this cross-sectional study are from the recruitment of a large study, a clinical trial that compared expulsion of postplacental copper IUD and the LNG 52mg intrauterine system (IUS). 14 Thus, the sample is intentional because it includes the necessary number of women invited to reach the sample size of the clinical trial.

The insertion of PPIUD was offered to women that would go through a cesarean delivery or were admitted in labor at the Women Hospital of the Universidade de Campinas. The exclusion criteria were the presence of any maternal infection or anemia, rupture of membranes for > 18 hours, uterine malformation, or twin pregnancy. Also, the pregnancy had to have been ≥ 37 weeks long and the parturient age had to be between 18 and 43 years old. If the parturient was classified as a candidate, PPIUD was offered. The present study was conducted between May 2018 and January 2019.

In case of acceptance, the patient was randomized to receive a TCu380A IUD or an LNG IUS, and a total of 70 units of each was inserted. In case of refusal, the woman was asked why she did not want PPIUD insertion. Subsequently, the refusal reasons were grouped according to misinformation or other reasons. Fear of pain, bleeding, contraception failure, and IUD impairing fertility were considered misinformation.

To evaluate if there was a statistical difference between the mean age of acceptance and refusal, the Mann-Whitney test was performed. Besides that, women who refused PPIUD were categorized in 2 age groups (between 18 and 27 years old versus between 28 and 43 years old) to analyze if the refusal motives showed any tendency (misinformation versus other reasons) among these groups (a χ ^2^ test was performed). Also, the mean age of the patients who refused PPIUD due to misinformation was compared with the mean age of the women who refused for other reasons, and a Mann-Whitney test was performed to evaluate if there was statistical significance. All information was analyzed by SAS Statistical Analysis System for Windows, version 9.2 (SAS Institute, Inc., Cary, NC, USA).

## Results

Postplacental placement of intrauterine devices was offered to 241 women, of whom 140 accepted PPIUD insertion (58.1%). Of all the patients involved, 74 were < 24 years old (30.7%), while 167 were between 25 and 43 years old (69.3%). There was no significance in the mean age of the patients who refused or accepted PPIUD insertion ( Table 1 ).

**Table 1:** Postplacental intrauterine device placement acceptance and refusal according to women age

	Acceptance		Refusal		
Women age (years old)	*n * = 140	%	*n* = 101	%	*p-value*
18–24	46	32.9	28	27.7	0.825 *
25–29	41	29.3	31	30.7	
30–34	30	21.4	22	21.8	
35–43	23	16.4	20	19.8	
Mean age/SD *	27.9 ± 5.8		28.05 ± 6.2		0.506 **

Abbreviation: SD, standard deviation.

* Chi-squared test

** Mann-Whitney test

**Fig. 1:**
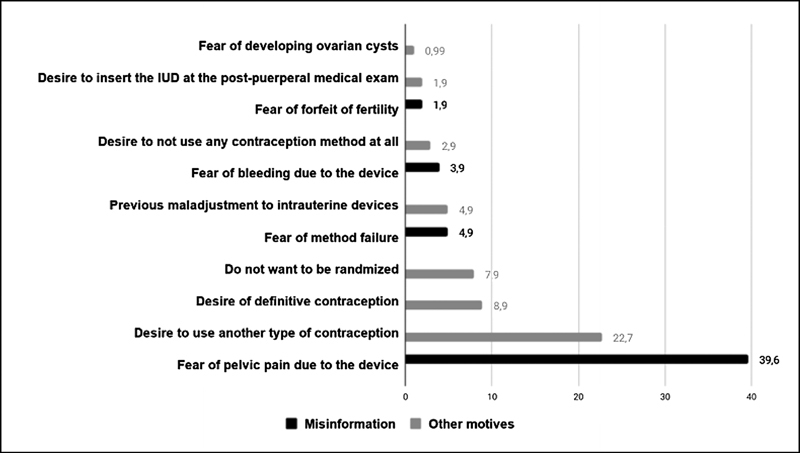
Motives for refusal of postplacental intrauterine device placement

The motives to refuse the PPIUD are described in Fig. 1 . To correlate the refusal motives with the knowledge of the patient about contraception methods, fear of pain, bleeding, contraception failure and IUD impairing fertility (in black) were considered examples of misinformation. All other motives (in gray) were considered as not being correlated with misinformation (desire for nonreversible contraception methods such as sterilization or vasectomy; desire to use another type of contraception; desire to not use any contraception method at all; previous maladjustment to IUDs; fear of developing ovarian cysts; desire to insert the IUD at the postpuerperal medical consultation; and desire to not be randomized). Therefore, 50.5% of all refusals were due to a lack of knowledge of contraception methods, that is, misinformation.

It was also observed that patients aged between 18 and 27 years old were more likely to refuse PPIUD insertion due to misinformation (67.4%) when compared with those aged between 28 and 43 years old (36.47%), who usually refused this type of contraception because of other reasons (63.6%), (chi-squared test; *p* = 0.002). There was difference between the mean age of the patients who did not want the PPIUD insertion due to lack of knowledge (27.3 ± 6 years old) and of those who did not want it due to other reasons (29.8 ± 5.8 years old), with statistical relevance (Mann-Whitney test; *p* = 0.017). In all ages, the refusal rate due to misinformation was high (67.4 and 36.47%, respectively). These data are summarized in Table 2 .

**Table 2:** Refusal of post placental intrauterine device by misinformation and women age

	Misinformation *n* = 51 (50.5%)		Other reasons *n* = 50 (49.5%)		
	Mean age	SD	Mean age	SD	p-value
	27.3	6.4	29.9	5.4	0.017*
**Age groups (years old)**	***n***	**%**	***n***	**%**	
18–27	31	60,78	15	30	0.002**
28–43	20	39,21	35	70	

*Mann-Whitney test

**Chi-squared test

## Discussion

Our study showed that the rate of refusal of IUDs immediately after childbirth was high (41.9%), mainly due to lack of information about IUD by the patients, especially amongst younger women.

The practice of PPIUD insertion has gained attention recently and is recommended by the World Health Organization (WHO). It should be considered an excellent contraceptive method, because women are rarely in such frequent and intense contact with health professionals as they are during pregnancy and the immediate postpartum period. Therefore, this period is a good opportunity to promote education and provide counseling in reproductive health.

After childbirth, it is common for recent mothers to develop many concerns about the newborn and often forget about their own health. The fact that 40% of all women who give birth do not attend puerperal appointments 9 reflects this loss of selfcare. Also, it is common for women after childbirth to think less about contraception, 15 and any period with no protection could result in an unintended pregnancy. 16 Since the antenatal visits will be the period when a woman has most frequent contact with a healthcare provider, it is also the duty of the healthcare provider to discuss the reproductive future of the woman. The period immediately after childbirth is a good opportunity to initiate contraception, including IUDs, because the patient is not pregnant, probably does not want to conceive in the near future, and will not feel pain during its insertion. It is important to study the acceptance rates of the use of contraception immediately after childbirth, as well as the motives for its refusal, in order to promote this type of family planning.

The present study showed that slightly more than half of the patients accepted PPIUD, and their mean age was 28 years old. The refusal rate was high (41.9%), and the most frequent refusal motives were misinformation about IUD, such as: fear of pain, bleeding, contraception failure and IUD impairing fertility. Intrauterine devices do not cause pelvic pain, prejudice fertility, or have a high failure risk. 6 17 18 As for bleeding, although copper IUDs may increase menstrual blood flow, this can be easily controlled with medication. These refusal motives can, therefore, be easily demystified, but only by properly counseling the patients about this method. Patients aged between 18 and 27 years old and between 28 and 43 years old refused PPIUD insertion frequently due to lack of information and, amongst the younger patients, the chances of refusing this contraception due to misinformation are higher.

The present study has some limitations. The sample size is intentional, based on the sample calculated for a randomized study. 14 Epidemiological data, such as race and education, were not collected. However, we consider the results obtained in this simple analysis very strong. Recognizing misinformation as a barrier to PPIUD use, especially amongst those of a young age, is the first step in the development of public policies on contraception that should be added to others such as training of healthcare professionals. Other studies performed in developing countries have also shown that the lack of IUD awareness impacts on low acceptance of this type of contraception and encourage policies to educate women about contraception and IUDs. 19 These studies agree that educating couples about contraception and antenatal care increases PPIUD usage. 20

As other studies, the present study shows that misinformation about contraception is frequent amongst women, and that this failure facilitates the occurrence of unintended pregnancies. 21 22 Also, basic interventions such as counseling increase IUD acceptance, 23 and multiple approaches on this matter enhance the rate of acceptance by women immediately after parturition. 24 A recent study showed that with PPIUD, almost all the expulsions occurred within 42 days after childbirth, and suggests special attention during this period to identify premature expulsions. 14 Postplacental placement of IUD or intrauterine system (IUS) is associated with less discomfort during the procedure; however, it is associated with higher expulsion rates than other interval placements. 25

The period of gestation is, therefore, an excellent moment to clarify with women the importance of contraceptive methods, the preference for long-term methods, the benefits of IUD, and the advantages of its insertion immediately after childbirth. Obstetricians and gynecologists and other health care agents should frequently talk to pregnant women about family planning. Other important information that should be shared with pregnant women is that contraception immediately after delivery improves perinatal outcomes for the woman herself and for the newborn – studies show an increase in child survival rates, a decrease in unintended pregnancies and maternal mortality, and a reduction in maternal depression. 26 27 28

## Conclusion

The present study has showed that the rate of refusal of IUDs after childbirth was high, mainly due to lack of information about the devices by the patients, especially amongst younger women. Policies need to be revised to increase contraception awareness after childbirth, through measures such as family planning groups with pregnant women, information sheets and counseling during prenatal care appointments.
